# Nichol’s Elixir

**Published:** 1879-11

**Authors:** 


Nichols' Elixir of Peruvian Bark with Protoxide of Iron, now manufactured by Messrs. Billings, Clapp & Co., of Boston, has a well-earned reputation among experionced physicians. It is especially adapted to cases requiring a bitter tonic combined with iron, who are in need of a pleasant, palatable and, at the same time, effective preparation.

